# Tailoring the Microstructure of Ti-6Al-4V Alloy Fabricated by Hybrid Additive Manufacturing via Rolling Strategies

**DOI:** 10.3390/ma19183812

**Published:** 2026-09-08

**Authors:** Jixin Yang, Zhiqin Yang, Xu Gu, Ying Bao, Huaping Tang

**Affiliations:** 1 Institute of Advanced Additive Manufacturing, Ji Hua Laboratory, Foshan 528200, China; guxu@jihualab.ac.cn (X.G.); tanghp@jihualab.ac.cn (H.T.); 2 Institute of Advanced Technology, Guangzhou International Campus, South China University of Technology, Guangzhou 511422, China; 3 School of Mechanical Engineering, Henan International Joint Laboratory of Rare Earth Composite Materials, Henan University of Engineering, Zhengzhou 451191, China; baoyinghit@outlook.com

**Keywords:** hybrid additive manufacturing, in situ rolling, titanium alloy, microstructure, globularization

## Abstract

In situ rolling-assisted laser directed energy deposition (IR-LDED) was employed to fabricate Ti-6Al-4V titanium alloy samples, focusing on the microstructural characteristics under various rolling strategies. The results indicate that the microstructure was primarily governed by the interplay between temperature-dependent deformation and recrystallization processes. In situ rolling at elevated temperature, combined with complex thermal cycling, promoted recrystallization within the deposited material, leading to significant grain refinement. After remelting and high-temperature rolling, the average grain size in the middle region of the sample was refined to 21 μm, accompanied by high kernel average misorientation (*KAM*_avg_) value of 1.92. Increasing the number of rolling passes at lower temperatures further reduced the grain size and decreased the *KAM*_avg_. Following in situ rolling, the deposited material underwent remelting, and additional rolling at elevated temperatures increased both deformation and *KAM*_avg_. The rearrangement of dislocations, formation of subgrain boundaries, and enhanced solute diffusion collectively facilitated the globularization of lamellar α phases, ultimately forming a microstructure composed of globular α, lamellar α/β, and dot-like β phases.

## 1. Introduction

Ti-6Al-4V alloy is extensively utilized across the aerospace, biomedical, and automotive industries due to its outstanding specific strength, corrosion resistance, and biocompatibility [1]. Laser directed energy deposition (LDED), characterized by high deposition efficiency and flexible printing strategies, offers significant advantages for the integral fabrication of large-scale critical components [2,3]. However, titanium alloy parts produced via LDED in their as-built state often suffer from issues such as low relative density, coarse grain structures, and anisotropic mechanical properties, which limit their application in load-bearing components [4].

To overcome these limitations, plastic deformation techniques have been incorporated into the LDED process, forming hybrid additive manufacturing (HAM) methodologies [5,6]. Colegrove et al. [7] employed inter-layer rolling to Ti-6Al-4V components produced by wire arc additive manufacturing, refining coarse β columnar grains into equiaxed grains, thereby reducing microstructural anisotropy and enhancing mechanical properties. Huang et al. [8] and Yang et al. [9,10] utilized in situ rolling-assisted LDED to fabricate alloys such as 316L stainless steel, Invar 36, and Ti-6Al-4V, achieving grain refinement, defect control, and performance enhancement. Xu et al. [11] fabricated 316L stainless steel components with heterogeneous microstructures via ultrasonic rolling-assisted LDED, inducing grain refinement and dislocation strengthening that substantially improved fracture toughness. Lv et al. [12] employed ultrasonic impact treatment to tailor the surface roughness, residual stress distribution, and tensile properties of Ti-6Al-4V titanium alloy components prepared by arc additive manufacturing. The ultrasonic impact transformed residual tensile stress in the surface layer into compressive stress, refined grains, and simultaneously enhanced both strength and ductility. Wu et al. [13] investigated the effect of synchronous hammer forging on the microstructure and mechanical properties of 316L stainless steel samples processed by LDED, achieving plastic deformation exceeding 21% with a relatively small load and reducing the average grain size by 69%. Navarro et al. [14] applied laser peening and shot peening to Ti-6Al-4V parts fabricated by selective laser melting (SLM), significantly enhancing fatigue strength through surface plastic deformation treatments. Overall, these plastic deformation-assisted approaches have demonstrated beneficial effects on the microstructure and mechanical properties of additively manufactured metallic materials [15,16].

In addition, laser surface remelting has been recognized as an effective technique for enhancing the surface integrity and mechanical performance of additively manufactured components [17,18,19]. This process smooths surface depressions through the combined effects of gravity and surface tension, thereby reducing surface roughness and improving the overall surface quality. Zhang et al. [20] employed dual-laser in situ remelting to enhance the mechanical properties of 316L stainless steel fabricated by SLM, observing that remelting randomized grain orientations and weakened texture along the build direction (BD). Xie et al. [21] combined in situ laser remelting with heat treatment to refine a GH3536 nickel-based superalloy produced by SLM. This HAM approach facilitated the transformation from columnar to equiaxed grains, improved grain orientation uniformity, and effectively reduced residual stress and dislocation density. Guan et al. [22] employed a sequential process involving LDED, laser remelting, and annealing heat treatment to modify the microstructure of Ti-6Al-4V components. The laser remelting step notably decreased the grain boundary stability and promoted the development of a homogeneous microstructure.

This study employed various rolling strategies to prepare Ti-6Al-4V titanium alloy samples via in situ rolling-assisted LDED (IR-LDED). The influence of remelting and the number of rolling passes on the microstructure of the as-built Ti-6Al-4V alloy samples were investigated, and the effects of deformation temperature on the dimensions, phase morphology, and grain structure were analyzed. These findings provide an experimental foundation for the AM of high-performance titanium alloy components.

## 2. Materials and Methods

**Figure 1:**
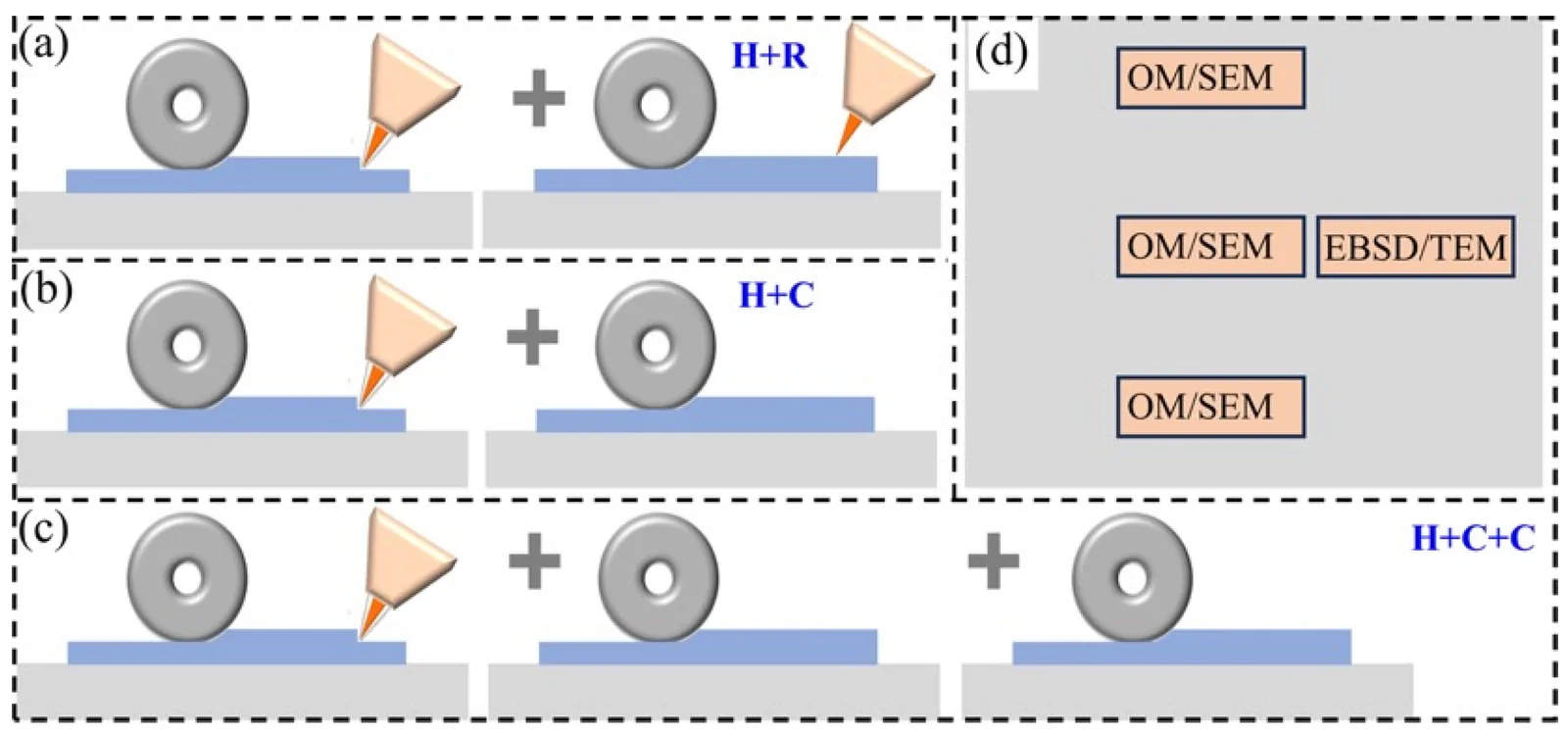
Schematic illustrations of the rolling strategies and sampling positions. (**a**) H+R; (**b**) H+C; (**c**) H+C+C; (**d**) schematic of the sampling positions.

Ti-6Al-4V samples were fabricated using an IR-LDED technique (developed by Ji Hua Laboratory, Foshan, China), with pre-alloyed powders produced by the plasma rotating electrode process (PREP) as the feedstock material. The particle size distribution was 53–150 μm, and the chemical composition was O 0.07, N 0.005, Al 6.21, V 4.03. The powder is spherical in shape, with an apparent density of 2.56 g·cm^−3^ and a flowability of 24.5 s/50 g, respectively. The rolling unit and deposition head were maintained in a fixed relative position throughout the process, with each layer deposited by controlling the horizontal movement of the machine tool. The roller is made of D2 steel, and the diameter and profile radius are 50 mm and 20 mm, respectively. Upon completion of a given layer, both the rolling unit and deposition head are simultaneously elevated by a distance (*L*) corresponding to the layer thickness to facilitate the deposition of the subsequent layer. Further details regarding the equipment configuration are available in a previously published study [23]. The processing parameters were set as follows: laser power of 1300 W, scanning speed of 480 mm/min, and an overlap ratio of 40%, while the rolling load was consistently maintained at 10 kN. The powder was delivered using high-purity argon as the carrier gas. The laser spot diameter was 3 mm, and the nozzle-to-substrate distance was 11 mm. A unidirectional scanning strategy was adopted, with the scanning direction rotated by 180° between adjacent layers. During the fabrication of the Ti-6Al-4V titanium alloy samples, three distinct rolling strategies were applied: (i) following in situ rolling, the powder feeding was ceased, and the rolling device returned to the starting point of the current pass to perform remelting and subsequent rolling (denoted as H+R; Figure 1a); (ii) after in situ rolling, the rolling device returned to the starting point and executed one additional rolling pass (denoted as H+C; Figure 1b); and (iii) subsequent to in situ rolling, the rolling device returned to the starting point and conducted two additional rolling passes (denoted as H+C+C; See Figure 1c). In the H+R process, the deposited layer is subjected to remelting before the subsequent rolling pass, which keeps the material at a significantly higher temperature than that in the H+C and H+C+C conditions. At this elevated temperature, the flow stress of Ti-6Al-4V decreases substantially, and therefore the same rolling load of 10 kN induces a larger plastic deformation, resulting in a thinner effective deposited layer. The corresponding layer lift values *L* for H+R, H+C, and H+C+C samples were 0.26 mm, 0.28 mm, and 0.28 mm, respectively. Without in situ rolling, the thickness of each layer was 0.50 mm, and the corresponding reduction ratios for the H+R, H+C, and H+C+C samples were 48%, 44% and 44%, respectively.

For microstructural characterization, specimens were sectioned parallel to the BD using wire electrical discharge machining. Following standard metallographic preparation, the specimens were etched with Kroll’s reagent. The microstructure was examined using optical microscopy (OM, Zeiss Stemi 508, Germany) and scanning electron microscopy (SEM, Thermo Fisher Verios 5 UC, Czech Republic). Mechanically polished samples were further subjected to vibratory polishing and then analyzed by electron backscatter diffraction (EBSD, Thermo Fisher Verios 5 UC, Czech Republic) to assess grain size and texture, and the acquired data were processed using Aztec Crystal software 2.1.2. EBSD scans were performed with a step size of 1.5 μm over an area of 0.8 mm × 0.8 mm, and the indexing rate exceeded 85%. The grain size was measured using the equivalent circle diameter method, and the average size of several hundred reconstructed grains within the scanned area was taken as the result. The grain orientation spread (GOS) was calculated for each grain, and grains were classified as follows: recrystallized grains with GOS < 1.5°, substructured grains with 1.5° ≤ GOS ≤ 3°, and deformed grains with GOS > 3°. Specimens extracted from the middle region of the as-built samples were ground to a thickness of 30 μm and then ion-milled. Dislocation morphologies of the samples subjected to various rolling strategies were investigated via transmission electron microscopy (TEM, Thermo Fisher Talos F200X G2, 200 kV, Czech Republic). The elemental distribution characteristics of Ti, Al, and V in the sample were obtained by energy-dispersive X-ray spectroscopy (EDS).

## 3. Results

### 3.1. Microstructure Characterization

**Figure 2:**
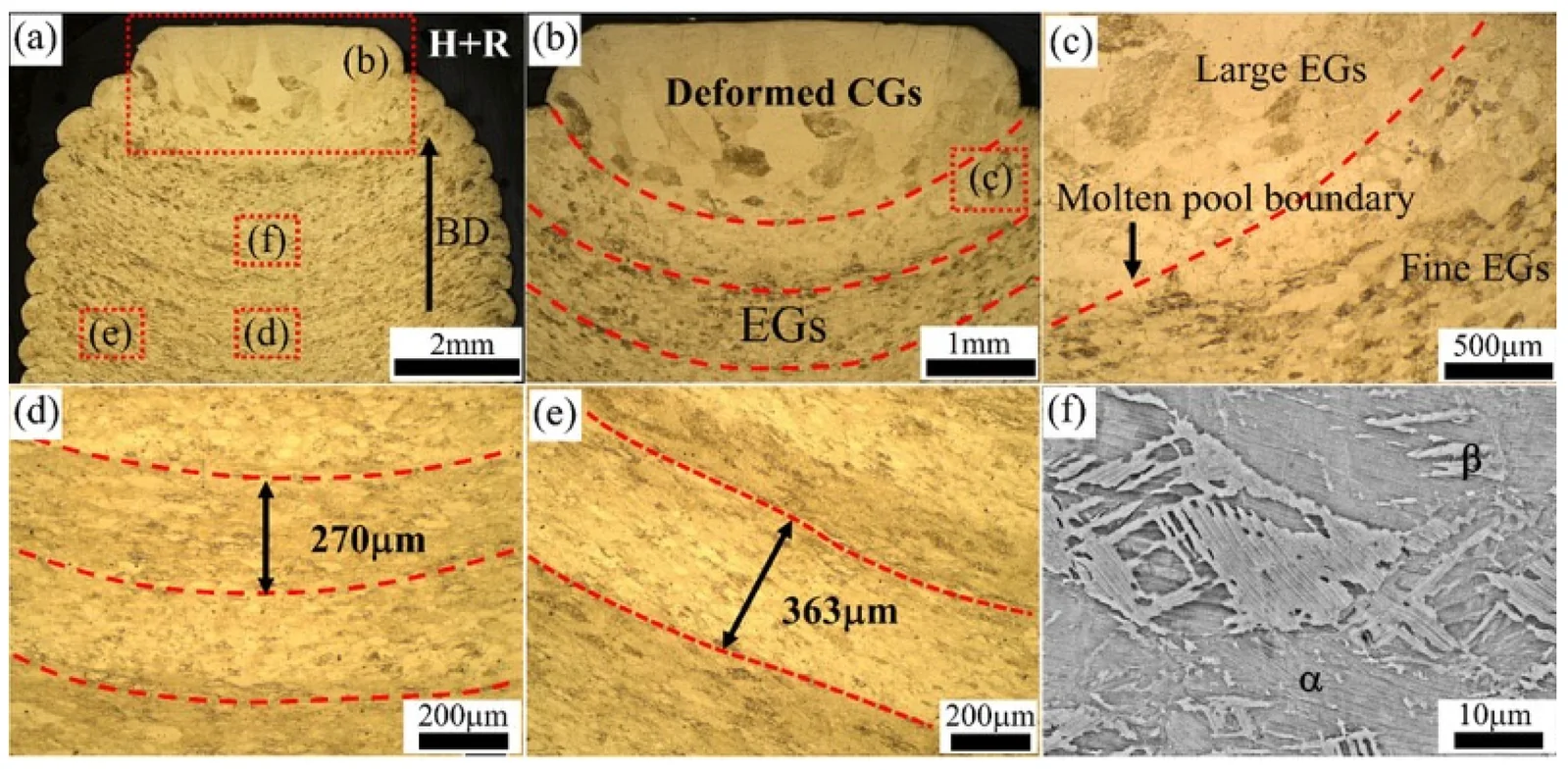
Microstructure of H+R samples fabricated by IR-LDED. (**a**) OM results of the cross-section parallel to the BD; (**b**) deformed CGs in the top region; (**c**) microstructure at the interface between the final layer and the penultimate layer; (**d**) fine EGs in the middle region; (**e**) grain morphology near the sample edge; (**f**) lamellar α/β phases. The red dashed line represents the molten pool boundary.

The cross-sectional microstructure of the H+R sample is shown in Figure 2. The sample’s upper surface exhibited a flat morphology, with wavy molten pool boundaries visible in the pre-solidified material (Figure 2a). Deformed columnar grains (CGs) were observed in the last layer, accompanied by fine equiaxed grains (EGs) located at the molten pool boundaries. The microstructure of the penultimate deposited layer consisted entirely of equiaxed grains, although the grain size was larger than that of the antepenultimate layer (Figure 2b,c). The melt pool spacing and single-wall thickness were determined by measurements at five different locations, and the average values were taken as the results. In the middle region of the sample, the spacing between molten pool boundaries of adjacent layers measured approximately 270 μm (Figure 2d), whereas near the sample edge, this spacing increased to 363 μm due to non-uniform pressure distribution during rolling (Figure 2e). The overall microstructure predominantly consisted of a lamellar α/β phase structure, as shown in Figure 2f.

**Figure 3:**
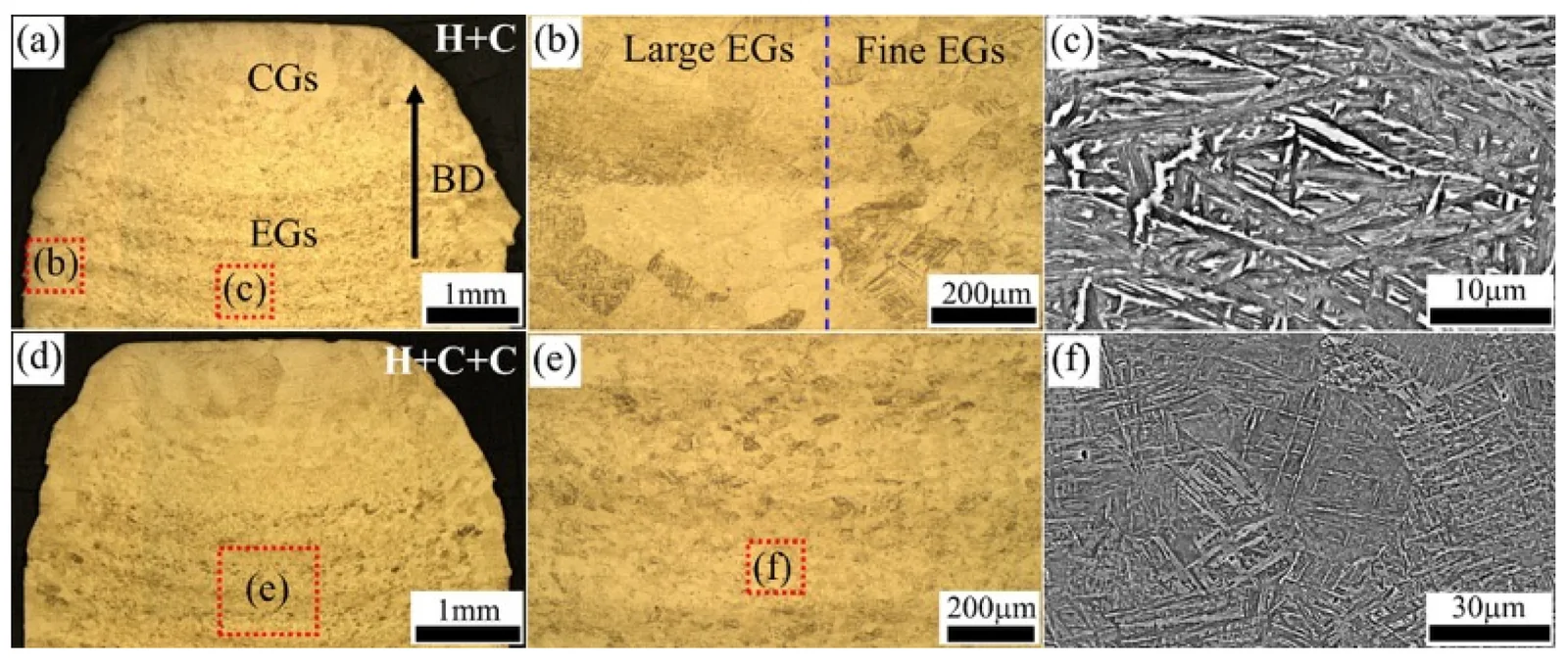
Microstructure of the H+C and H+C+C samples fabricated by IR-LDED. (**a**) OM results of the H+C sample cross-section parallel to the BD; (**b**) grain morphology close to the edge region; (**c**) SEM image showing lamellar α/β phases; (**d**) OM results of the H+C+C sample cross-section parallel to the BD; (**e**) grain morphology of the central region; (**f**) SEM image.

The cross-sectional microstructures of the H+C and H+C+C samples are shown in Figure 3. The outermost layer of both samples consisted of deformed CGs (Figure 3a,d). Near the edge, a heterogeneous microstructure featuring a combination of large EGs and fine EGs was observed, with grain size gradually decreasing toward the central region (Figure 3b,e). The microstructural characteristics of the H+C sample closely resembled those of the H+C+C sample, both predominantly consisting of lamellar α/β phases (Figure 3c,f). The measured widths of the single-wall samples H+C, H+C+C, and H+R were 5.1 mm, 5.7 mm, and 8.1 mm, respectively. Notably, the H+R sample exhibited a pronounced stepped morphology on its side surface, attributed to the elevated heat input and intense plastic deformation experienced during processing.

### 3.2. EBSD Results

**Figure 4:**
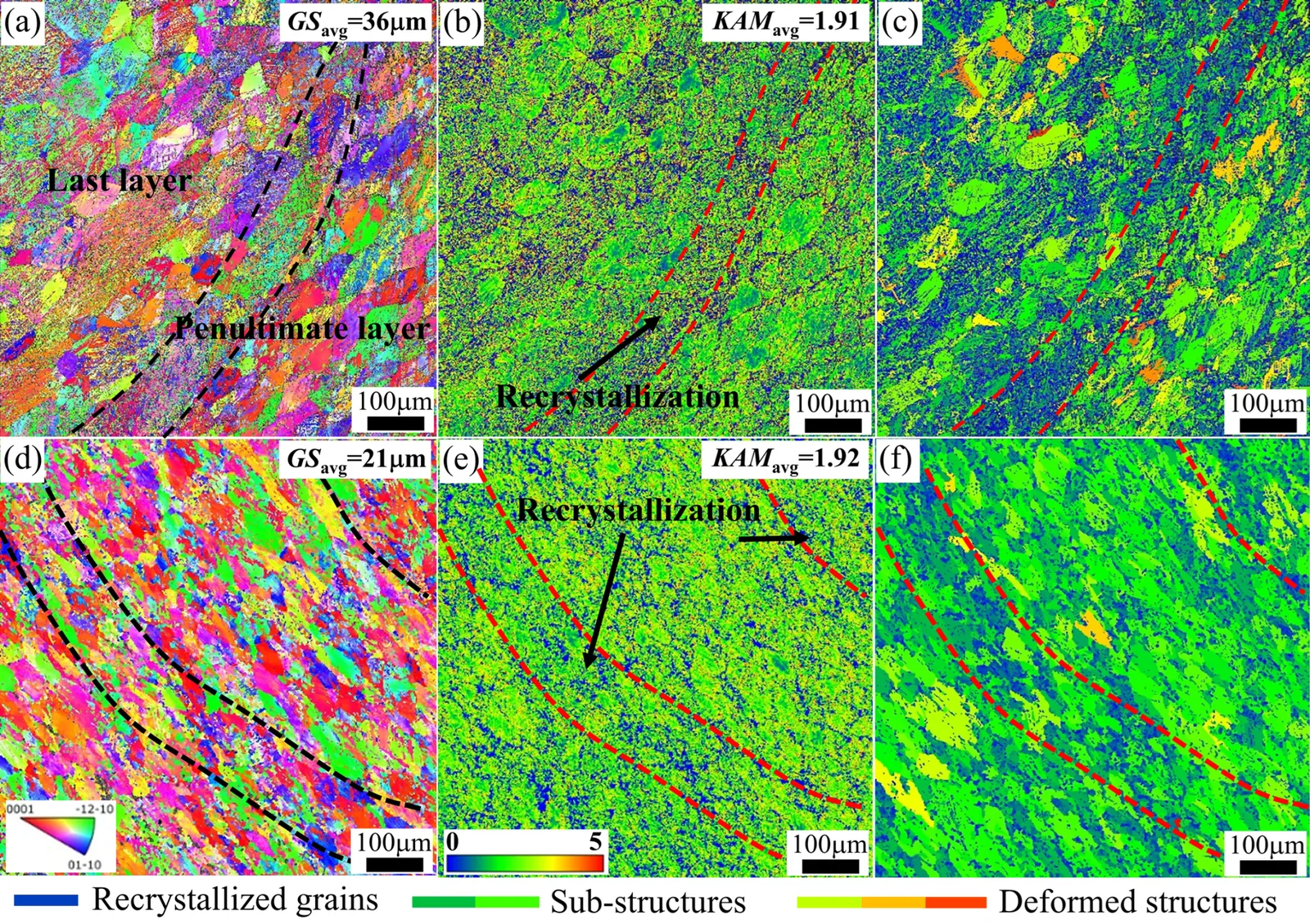
EBSD results of H+R samples fabricated by IR-LDED: (**a**–**c**) the top region; (**d**–**f**) the middle region; (**a**,**d**) inverse pole figures (IPF) maps; (**b**,**e**) KAM maps; (**c**,**f**) GOS maps. The dashed line represents the molten pool boundary.

The EBSD results for the top and middle regions of the H+R sample are presented in Figure 4. In the uppermost layer adjacent to the free surface, deformed CGs were observed, while the penultimate layer exhibited an EG morphology (Figure 4a). The average kernel average misorientation (*KAM*_avg_) and average grain size (*GS*_avg_) values were obtained from the scanned area, which contained hundreds of grains. The *GS*_avg_ in the top region was measured to be 36 μm. Due to plastic deformation, significant strain accumulation occurred in the last and penultimate layers, resulting in a *KAM*_avg_ value of 1.91 in the top region (Figure 4b). The *GS*_avg_ and *KAM*_avg_ values were derived from a single EBSD scan of a representative area of the sample. At the molten pool boundaries, recrystallization contributed to a decrease in the *KAM*_avg_ value, with the presence of recrystallized grains and substructured grains evident from the GOS maps presented in Figure 4c. In the middle region of the sample, the *GS*_avg_ was considerably refined to 21 μm, accompanied by a substantial presence of equiaxed α phases (Figure 4d). The *KAM*_avg_ value in the middle region did not decrease following recrystallization, suggesting that the previously deposited material experienced additional deformation after recrystallization (Figure 4e). This observation was further confirmed by the GOS map, which revealed an increase in substructures and deformed grains, as shown in Figure 4f.

**Figure 5:**
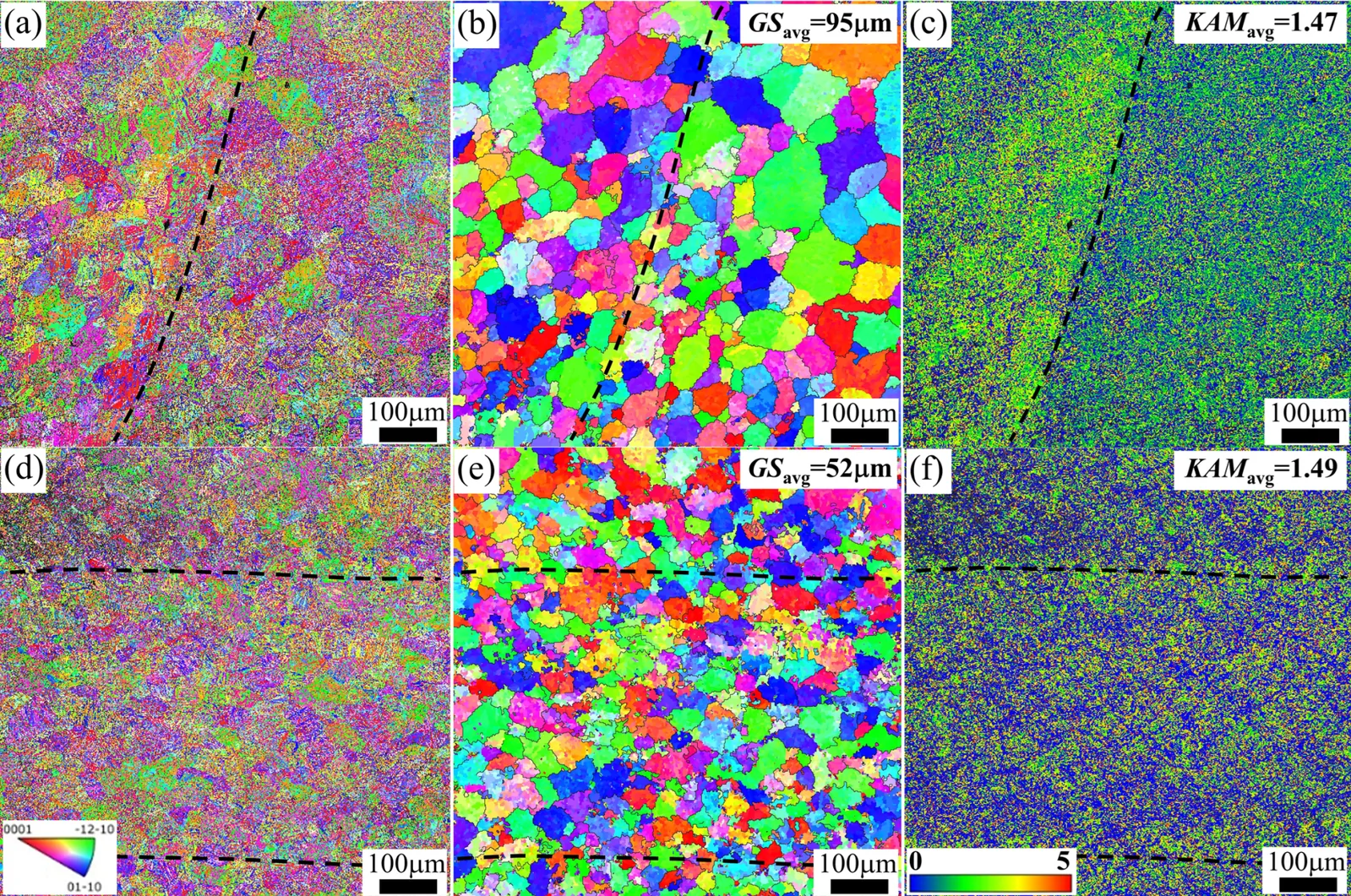
EBSD results of H+C samples fabricated by IR-LDED: (**a**–**c**) the top region; (**d**–**f**) the middle region; (**a**,**d**) IPF maps; (**b**,**e**) restructured grains; (**c**,**f**) KAM maps. The dashed line represents the molten pool boundary.

The EBSD results for the top and middle regions of the H+C sample are shown in Figure 5. Coarse EGs were observed near the free surface, with a *GS*_avg_ of 95 μm (see Figure 5a,b). In the pre-solidified layers, recrystallization caused a reduction in the *KAM*_avg_ value, and consequently, the *KAM*_avg_ value of the last layer was higher than that of the penultimate layer (Figure 5c). In the middle region of the sample, the *GS*_avg_ measured 52 μm, which was smaller than that observed in the top region (Figure 5d,e)), while the corresponding *KAM*_avg_ value was comparable to that of the top region (Figure 5f).

**Figure 6:**
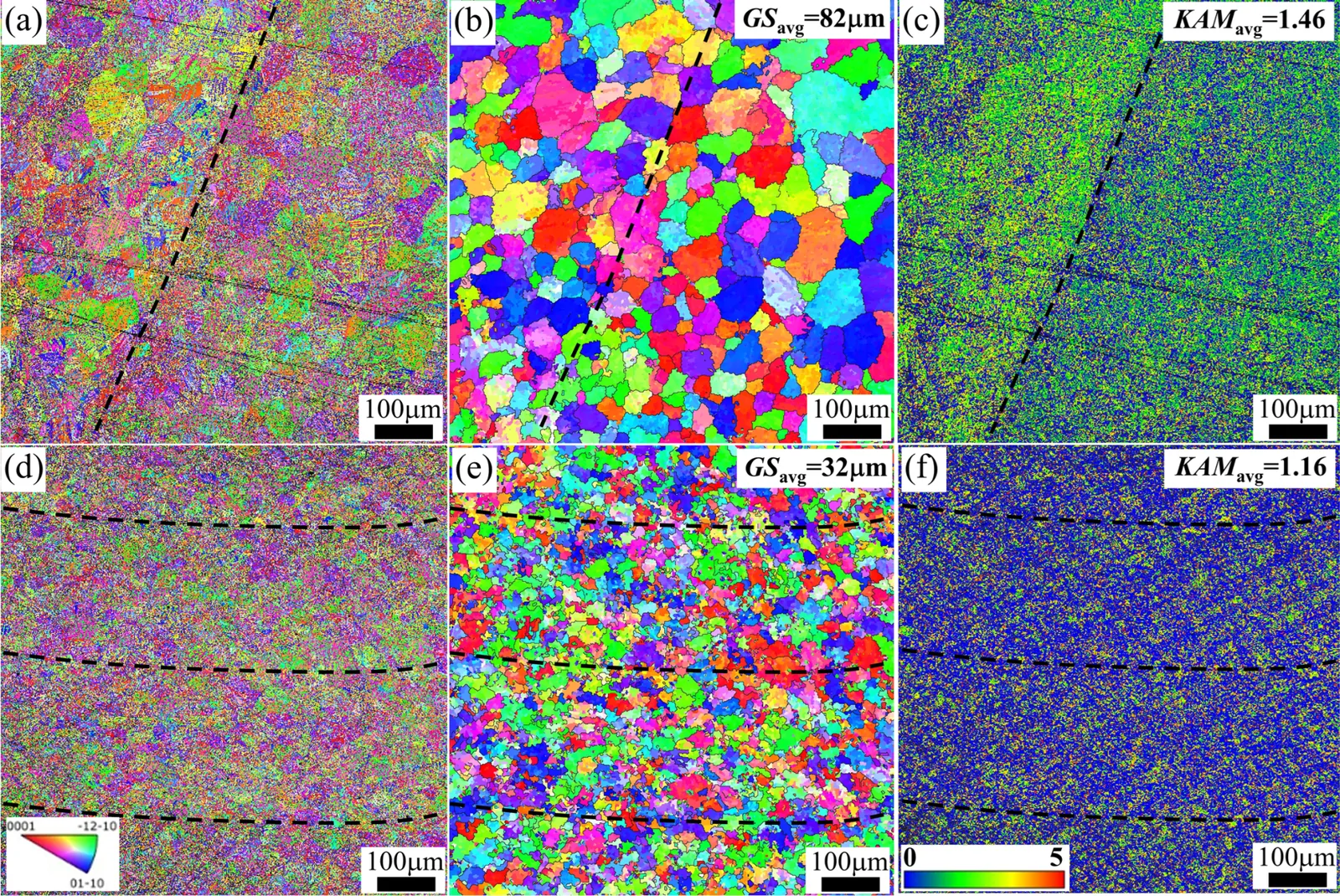
EBSD results of H+C+C samples fabricated by IR-LDED: (**a**–**c**) the top region; (**d**–**f**) the middle region; (**a**,**d**) IPF maps; (**b**,**e**) restructured grains; (**c**,**f**) KAM maps. The dashed line represents the molten pool boundary.

**Figure 7:**
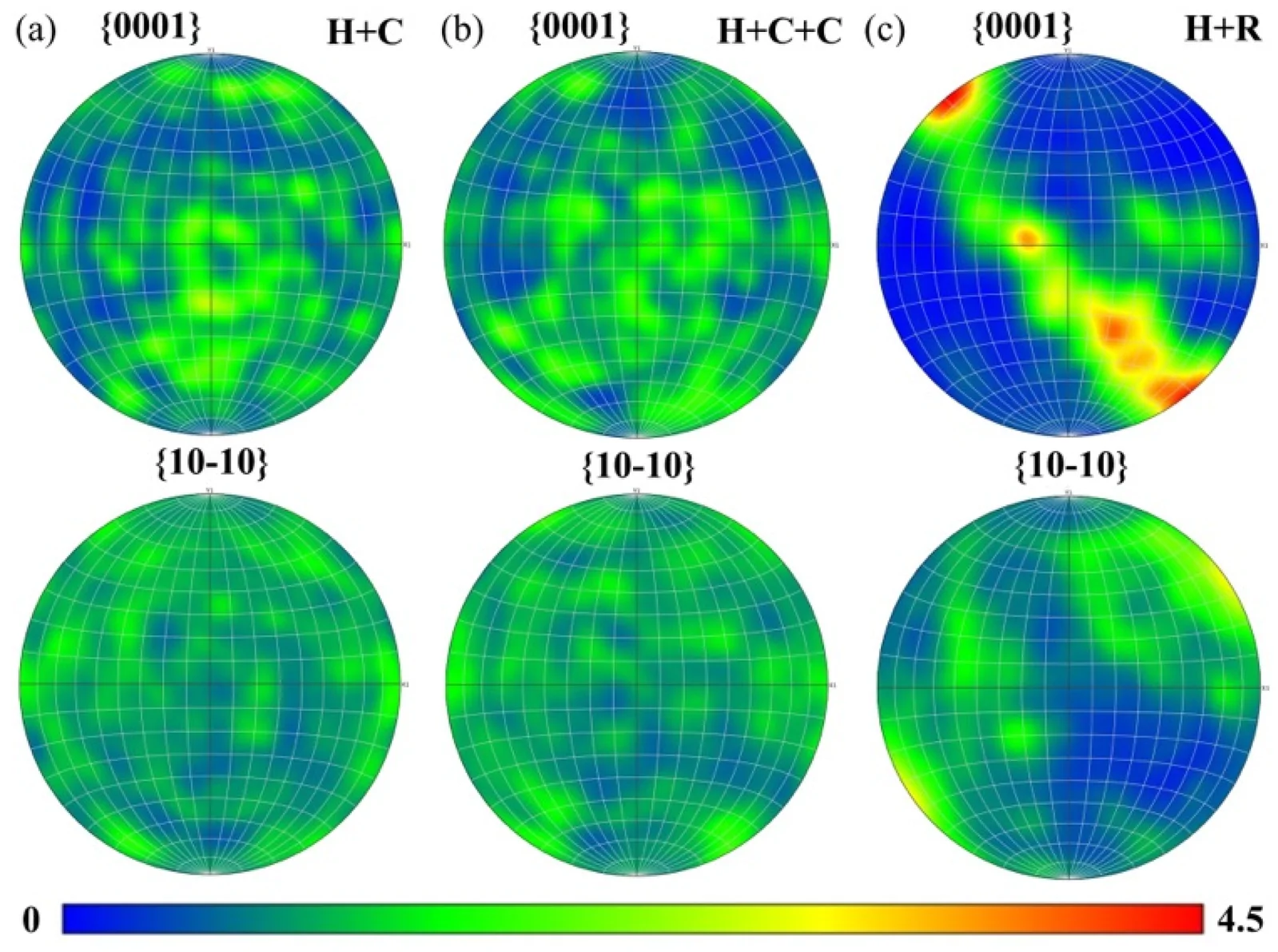
Pole figure of Ti-6Al-4V titanium alloy samples fabricated by IR-LDED: (**a**) H+C sample; (**b**) H+C+C sample; (**c**) H+R sample.

The EBSD results for the top and middle regions of the H+C+C sample are presented in Figure 6. Coarse EGs were observed in the top region (Figure 6a,b), whereas the middle region contained finer EGs (Figure 6d,e). The *GS*_avg_ values for the top and middle regions were 82 μm and 32 μm, respectively, with corresponding *KAM*_avg_ values of 1.46 and 1.16 (Figure 6c,f). The texture intensity of samples fabricated under different rolling strategies is shown in Figure 7, which were obtained from a single scan. The maximum texture intensities for the H+C and H+C+C samples were 2.65 and 2.21 (Figure 7a,b), respectively, both of which were lower than that of the H+R sample (Figure 7c). This reduction in texture intensity is beneficial for reducing the mechanical anisotropy of the samples.

### 3.3. TEM Results

**Figure 8:**
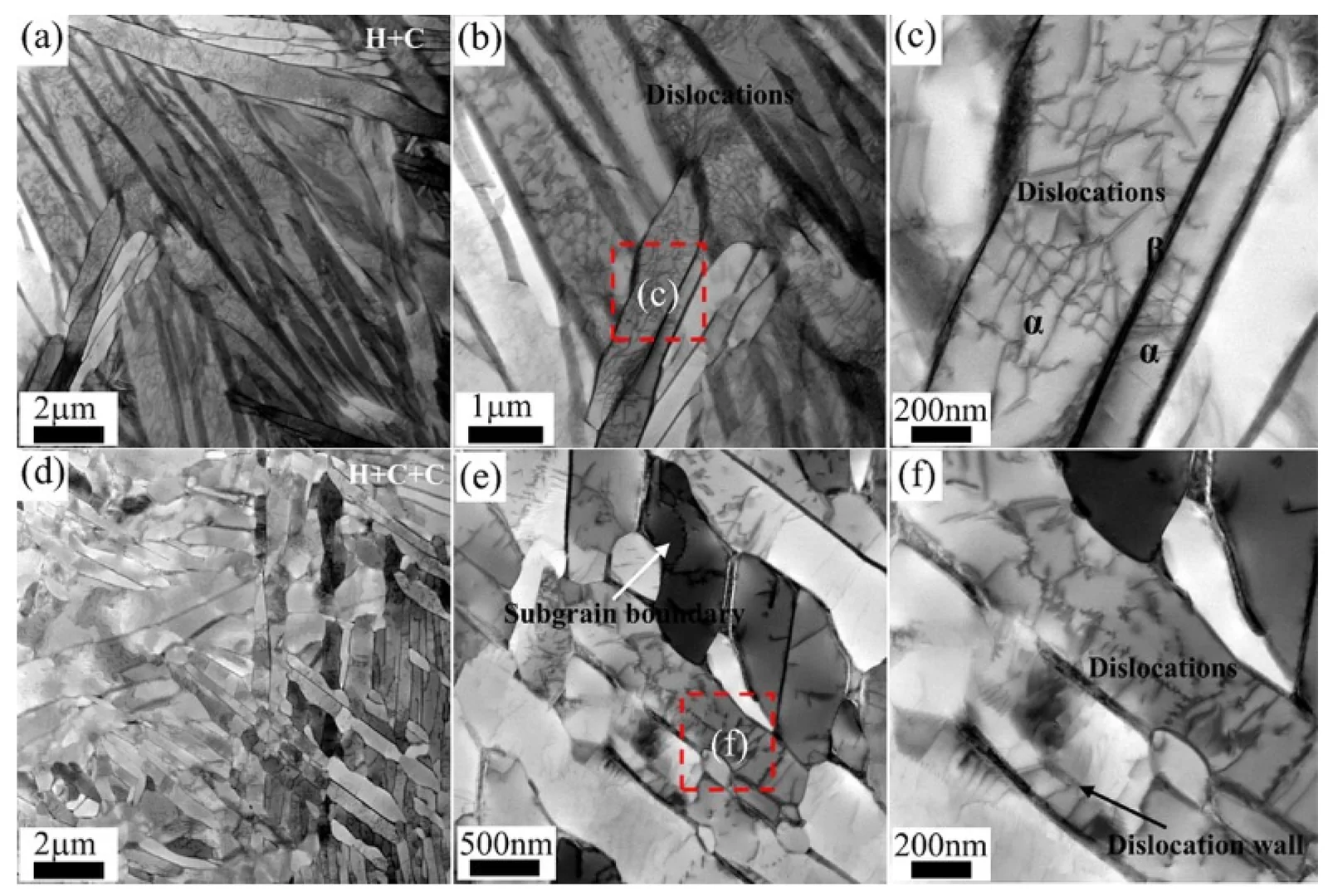
TEM results of H+C and H+C+C samples fabricated by IR-LDED: (**a**–**c**) H+C samples; (**d**–**f**) H+C+C samples; (**a**) lamellar α/β phases; (**b**,**c**) dislocation tangles; (**d**) lamellar and globular α phases; (**e**) subgrain boundary; (**f**) dislocation wall.

The TEM results of the H+C and H+C+C titanium alloy samples are shown in Figure 8. The H+C sample exhibited a lamellar α/β microstructure (Figure 8a), with a high density of entangled dislocations visible within the α phases due to plastic deformation of the deposited material (Figure 8b,c). In the H+C+C sample, both a lamellar α/β structure and globular α phases were observed, with subgrain boundaries apparent within some lamellar phases (Figure 8d). Additionally, dislocation walls (DWs) alongside entangled dislocations were observed, and fragmentation of a minor fraction of lamellar α phases was noted (Figure 8e,f).

**Figure 9:**
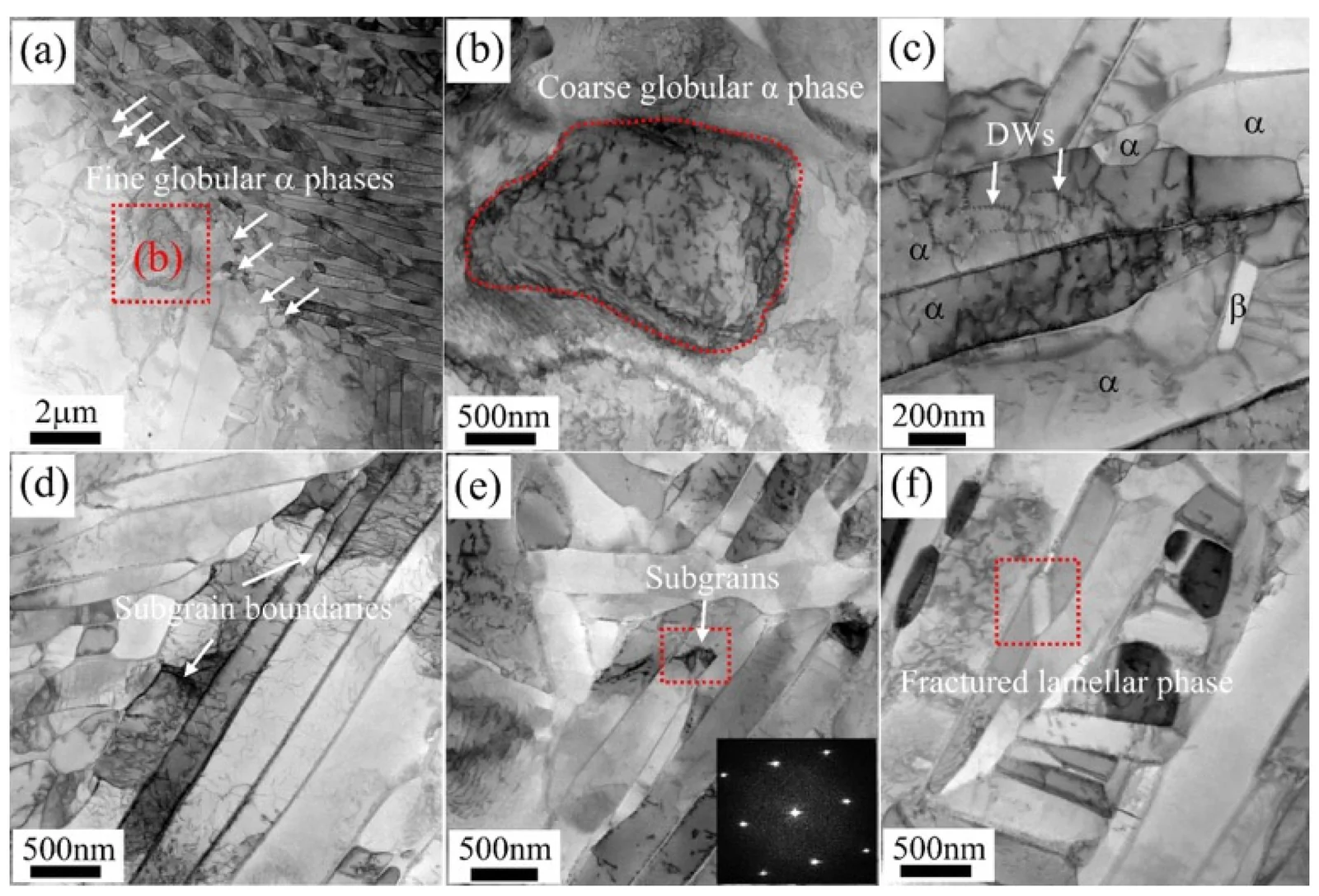
TEM results of H+R samples fabricated by IR-LDED. (**a**) Bright-field image showing lamellar α phases, coarse globular α phases, and fine globular α phases; (**b**) the coarse globular α phases; (**c**) DWs; (**d**,**e**) subgrain boundaries within the lamellar α phases with a high aspect ratio; (**f**) fractured lamellar α phases.

TEM results of the H+R titanium alloy samples are presented in Figure 9. Coarse globular α phases and lamellar α phases were observed in the H+R sample, with fine globular α phases localized at the boundaries of the molten pool (Figure 9a). A substantial number of dislocations were detected within the coarse globular α phases, and DWs were apparent within the lamellar α phases, as shown in Figure 9b,c. Certain lamellar α phases exhibiting a high aspect ratio contained subgrain boundaries and underwent progressive fragmentation into multiple segments, as shown in Figure 9d–f. The zone axis of the diffraction pattern in Figure 9e was $\left[11\bar{2}0\right]$.

**Figure 10:**
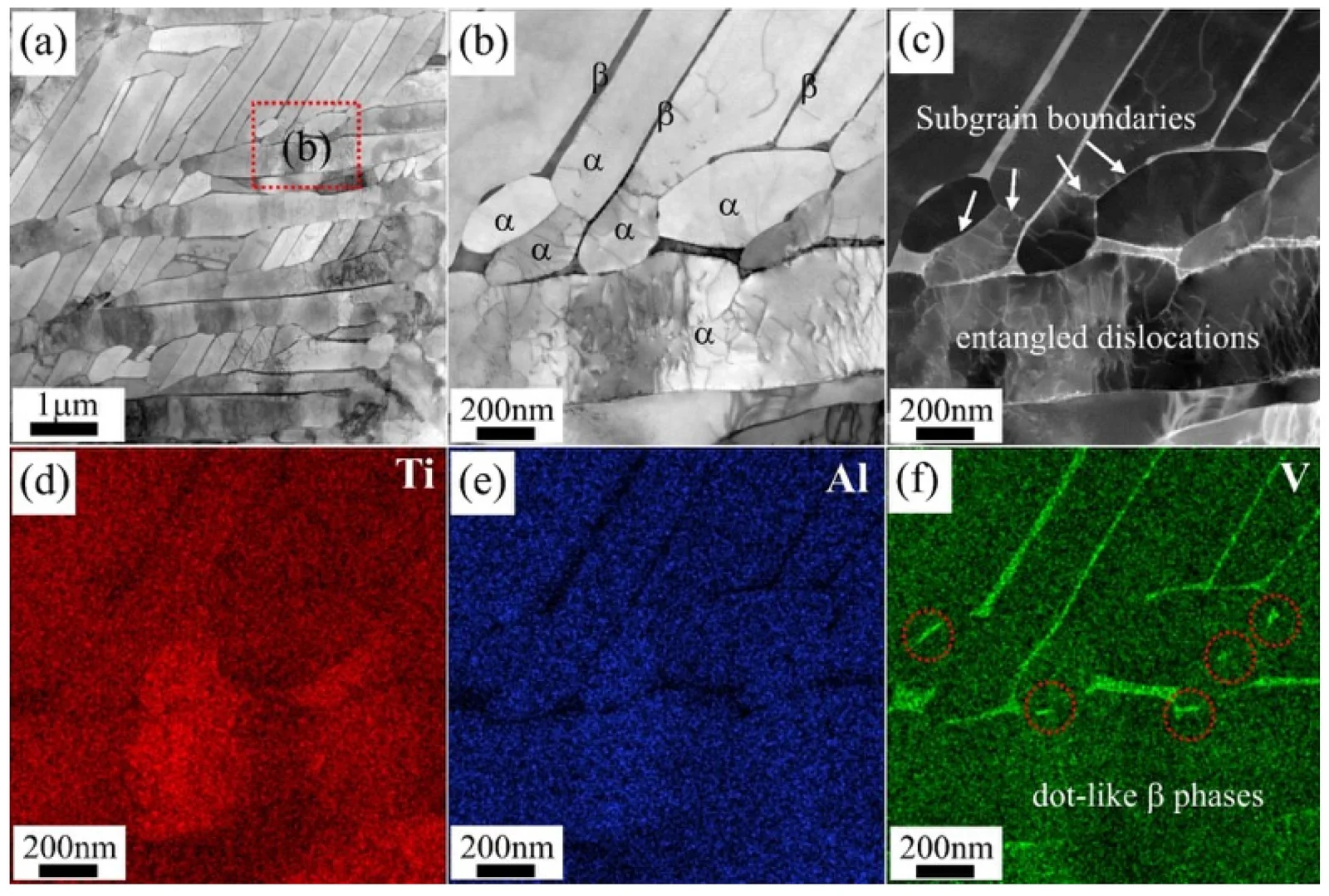
Elemental distribution results of H+R samples fabricated by IR-LDED. (**a**,**b**) Lamellar and globular α phases; (**c**) dark-field image showing subgrain boundaries and tangled dislocations; mapping result of (**d**) Ti; (**e**) Al; (**f**) V.

Figure 10 illustrates the morphological characteristics and elemental distribution of the globular and lamellar α phases within the H+R sample. Numerous globular α phases were observed throughout the sample, while the lamellar α phases exhibited an increased thickness and a decreased aspect ratio, as shown in Figure 10a. Entangled dislocations were present within the lamellar α phases, and the globular α phases were subdivided into subgrains delineated by subgrain boundaries, as depicted in Figure 10b,c. Furthermore, the originally continuous lamellar β phases transformed into discrete, dot-like β phases due to elemental diffusion, as shown in Figure 10d–f.

## 4. Discussion

The variations observed among the three rolling strategies can be attributed to the interplay of thermal input, incremental strain accumulation across layers, and the extent of recrystallization, all of which critically influenced the microstructural characteristics of the as-built samples. Based on the previous temperature monitoring results under similar processing conditions [9], it can be inferred that the deposited material in the H+C and H+C+C samples underwent high-temperature deformation at approximately 550 °C while the temperature had dropped below 300 °C during the subsequent re-rolling pass. In contrast, owing to the higher heat input in the H+R sample, the temperature of the deposited material during both deformation events remained above 550 °C. Given that infrared measurements represent an average temperature over a 3 mm diameter area, the actual temperature at which plastic deformation occurs within the deposited material is likely higher. Consequently, the deposited material initially undergoes plastic deformation at approximately 550 °C, followed by one or two additional deformation passes at temperatures below 300 °C. In contrast, the H+R sample, benefiting from supplementary heat input due to remelting, experiences plastic deformation at an elevated temperature, resulting in greater deformation under an equivalent rolling load. The introduction of the remelting process fundamentally alters the thermal history, solidification history, deformation history, and phase morphology.

In the H+R sample, the deposited material undergoes plastic deformation following the initial in situ rolling pass, but the layers previously deformed during the preceding pass are eliminated through interlayer remelting. CGs form after the rapid solidification of the molten pool [24,25]. Subsequently, the solidified material experiences further deformation at elevated temperatures, resulting in the development of deformed CGs. The elevated temperature in the H+R strategy lowers the flow stress of Ti6Al4V, so the same rolling load of 10 kN produces a larger plastic deformation than in H+C and H+C+C. This plastic deformation results in a high *KAM*_avg_ value in the final layer, while the *KAM*_avg_ value decreases at the molten pool boundary due to recrystallization. In the penultimate layer, reheating combined with deformation induces partial transformation of these deformed CGs into EGs. As the layer-by-layer deposition progresses, the residual deformed CGs also undergo recrystallization under repeated thermal cycling and accumulated deformation, which explains the smaller *GS*_avg_ observed in the middle region compared to the top region. At high temperature, enhanced thermal activation promotes dislocation movement, and dislocations in the lamellar α phases progressively organize into planar dislocation arrays through further slip, climb, and rearrangement. These arrays subsequently form subgrain boundaries via polygonization, characterized by unstable dihedral angles [26,27]. Dislocations act as high-diffusivity pathways that enhance atomic diffusion, and solute diffusion driven by curvature differences at phase interfaces further accelerates morphological changes in the lamellar phases, ultimately leading to the formation of thermal etching grooves. This phenomenon eliminates internal phase boundaries and establishes new boundaries, thereby promoting microstructural globularization [28,29].

Defects such as lamellar boundaries and terminations of certain lamellar α phases with high aspect ratios compromise microstructural stability. Under elevated temperatures, the lamellar structure undergoes fragmentation accompanied by rapid elemental diffusion. The lamellar phases are intersected by precipitated β phases, forming a globular morphology characterized by a low aspect ratio [30,31]. In the H+R samples, the presence of fine, uniform equiaxed grains alongside numerous globular α phases effectively accommodates strain during deformation and delays crack initiation. Furthermore, the coarsening and globularization of lamellar α phases, coupled with the transformation of β phases into a dot-like morphology, reduce the interfacial area between phases and mitigate stress concentration. These microstructural evolutions facilitate plastic deformation and contribute to an enhanced strength–ductility synergy [32].

The H+C and H+C+C samples undergo deformation at comparable temperatures, resulting in similar final microstructures. Following in situ rolling, the number of dislocations within the deposited material increases, with further multiplication of dislocations occurring during one or two subsequent rolling passes. However, the relatively low deformation temperature impedes dislocation climb and cross-slip, leading to the retention of a substantial number of entangled dislocations within the α phases. During thermal cycling, these entangled dislocations rearrange into DWs and further develop into subgrain boundaries characterized by larger misorientation angles. The increased degree of deformation facilitates recovery and recrystallization processes, thereby releasing local strain energy and resulting in a *GS*_avg_ in the middle region that is smaller than that observed in the top region [33]. Furthermore, the *KAM*_avg_ values in the middle region of the H+C sample exceeded those of the H+C+C sample, and grain size progressively refined toward the sample center along the horizontal axis. This phenomenon is attributed to the compression of the deposited material toward both sides during in situ rolling, which imposes the highest stress on the central region, thereby inducing the most pronounced grain refinement [34,35]. After in situ rolling, the texture intensities of the samples remained relatively low. However, the H+R sample exhibited a higher texture intensity compared to the H+C and H+C+C samples, attributable to the preferred crystallographic orientation introduced by directional solidification following remelting. Nevertheless, its texture intensity remained significantly lower than that typically observed in titanium alloys produced via LDED [36,37].

In summary, the formation of entangled dislocations, DWs, and subgrain boundaries is attributed primarily to the plastic deformation imposed by in situ rolling, which is particularly pronounced in the H+C and H+C+C samples where lower deformation temperatures restrict dislocation climb and cross-slip. The elimination of prior deformation history and the resetting of the solidification microstructure are direct consequences of the remelting step in the H+R strategy, which also enables subsequent deformation to occur at a higher temperature, thereby more effectively activating recovery and recrystallization. In addition, the progressive evolution of dislocation substructures, the partial recrystallization of deformed columnar grains, and the refinement of grain size from the top to the central region are associated with repeated thermal cycling and cumulative deformation during layer-by-layer deposition, especially in the H+C+C sample.

## 5. Conclusions

In this study, Ti-6Al-4V titanium alloy samples were fabricated using the IR-LDED process under three different rolling strategies. The microstructural evolution was investigated, and the main conclusions can be drawn as follows:

(1) The plastic deformation induced by in situ rolling promotes recrystallization, producing titanium alloy samples with equiaxed grain structures. The microstructure of the H+R sample comprised globular α phases, lamellar α/β phases, and discrete dot-like β phases. After remelting and high-temperature rolling, the *GS*_avg_ in the middle region of the H+R sample was refined to 21 μm, accompanied by a high *KAM*_avg_ value.
(2) The microstructures of the H+C and H+C+C samples consisted of lamellar α/β phases. The application of an additional rolling pass increased deformation within the deposited material, resulting in finer grain sizes and reduced texture intensity in the H+C+C sample compared to the H+C sample. In contrast, the H+R sample exhibited relatively high texture intensity, attributed to directional solidification following remelting.
(3) In situ rolling at high temperatures followed by rolling at a lower temperature resulted in the retention of a large number of entangled dislocations within the lamellar α phases in the H+C samples. An additional rolling pass further increased the number of dislocations in the H+C+C sample, with these dislocations reorganizing into DWs and subgrain boundaries during complex thermal cycling. In the H+R samples, high-temperature deformation combined with enhanced elemental diffusion promoted coarsening and globularization of the lamellar α phases and transformed the continuous lamellar β into dot-like β phases.

## Data Availability

The raw data supporting the conclusions of this article will be made available by the authors on request.
